# [Retracted] MicroRNA‑539 inhibits the proliferation and invasion of bladder cancer cells by regulating IGF‑1R

**DOI:** 10.3892/mmr.2024.13240

**Published:** 2024-05-13

**Authors:** Guiyi Liao, Fangmin Chen, Jinbiao Zhong, Xinan Jiang

Mol Med Rep 17: 4917–4924, 2018; DOI: 10.3892/mmr.2018.8497

Following the publication of this paper, it was drawn to the Editor's attention by a concerned reader that certain of the Transwell invasion assay data shown in Fig. 2C on p. 4921 were strikingly similar to data that had already been submitted for publication in different form in another article written by different authors at a different research institute.

Owing to the fact that the contentious data in the above article had already been published prior to its submission to *Molecular Medicine Reports*, the Editor has decided that this paper should be retracted from the Journal. The authors were asked for an explanation to account for these concerns, but the Editorial Office did not receive a reply. The Editor apologizes to the readership for any inconvenience caused.

